# Nivolumab to pembrolizumab switch induced a durable melanoma response: A case report

**DOI:** 10.1097/MD.0000000000013804

**Published:** 2019-01-11

**Authors:** Tanja Lepir, Mehdi Zaghouani, Stéphane P. Roche, Ying-Ying Li, Miguel Suarez, Maria Jose Irias, Niramol Savaraj

**Affiliations:** a Department of Veteran Affairs, Bruce W. Carter VA Medical Center, Miami, FL; b Florida Atlantic University, Department of Chemistry and Biochemistry, Boca Raton, FL; c Center for Molecular Biology and Biotechnology at Florida Atlantic University, Boca Raton, FL.

**Keywords:** melanoma, molecular interaction, PD-1 epitopes, PD-1/PD-L1 blockers

## Abstract

**Rationale:**

While checkpoint inhibitors have revolutionized the treatment of melanoma, it is not known whether switching from one monoclonal antibody drug to another one would be justified in the case of a treatment failure. Herein, we report a case illustrating a durable response to pembrolizumab after a failure with nivolumab.

**Patient concerns:**

A 76-year-old white male noticed an enlarging papular lesion on his neck.

**Diagnosis:**

Malignant melanoma.

**Interventions:**

The patient underwent surgery in December 2013 and was found to have a B-Rapidly Accelerated Fibrosarcoma (BRAF) V600E mutated melanoma. Treatment with BRAF and MAPK/Erk kinase (MEK) inhibitors along with radiation was initiated. After 1 year, the disease progressed, and the treatment was switched to the cytotoxic T-lymphocyte antigen 4 (CTLA-4) blocking antibody, ipilimumab. As the tumor did not respond, the treatment was changed to programmed cell death receptor-1 (PD-1) blockers: nivolumab followed by pembrolizumab. Since the initial diagnosis, the tumor response was monitored by computed tomography (CT) scans. Immunohistochemistry (IHC) was also used for the assessment of programmed death ligand 1 PD-L1) expression in the neck, lung, and spleen lesions.

**Outcomes:**

The patient had an initial mixed response to nivolumab, but the disease ultimately progressed as evidenced by new metastases to the spleen, thus the treatment was switched to pembrolizumab. After 46 cycles of treatment, all sites of metastases disappeared, including a substantial shrinkage of the splenic metastasis. To gain understanding about the pharmacological differences between nivolumab and pembrolizumab, the PD-1–ligands interactions and conformational dynamics responsible for the PD-1/PD-L1 checkpoint blockade were investigated. The higher affinity of pembrolizumab might likely arise from a unique and large patch of interactions engaging the C’D loop of PD-1, thus forcing an important motion across the PD-1 immunoreceptor.

**Lessons:**

In this case report, we described the tolerance and response of a melanoma patient to a sequence of various agents, including ipilimumab, nivolumab, and pembrolizumab. To the best of our knowledge, this is the first clinical report highlighting differences between PD-1 blockers, as shown by the unexpected and durable response of the tumor to pembrolizumab, after a treatment failure with nivolumab.

## Abstract 2

Supplemental Digital Content is available in the text

## 1 Introduction

It is known that 50–60% of melanomas harbor activating BRAF mutation^[1,2]^ and hence respond to BRAF inhibitors (BRAFi), but the duration of response is often short, despite adding MEK inhibitor to circumvent BRAFi resistance. While kinase inhibitors target specific BRAF mutations and have demonstrated response rate around 50–70%, resistance to treatment ultimately develops with approximately 85–90% of patients eventually relapsing within 1 year.^[3]^ On the other hand, immune checkpoint blockage with CTLA-4 inhibitor (ipilimumab), and PD-1 inhibitors (nivolumab or pembrolizumab) offer a longer duration of response and have brought dramatic improvements in the treatment of melanoma regardless of the mutation status.^[4,5]^ However, cancer immunotherapy drugs have limited efficacy in rapidly progressing disease because of their delayed onset of response. Importantly, we still do not know the best treatment sequence and what are the main structural, functional, and clinical differences between currently approved checkpoint inhibitors.

Immune checkpoints are important for maintaining self-tolerance and tempering the physiologic immune responses in peripheral tissues, therefore, they have recently drawn considerable interest in cancer immunotherapy.^[5]^ Indeed, tumors have been shown to exploit certain immune-checkpoint mechanisms to evade surveillance and escape the immune response. The PD-1 transmembrane protein receptor found in lymphocytes and monocytes pairing to its natural ligand PD-L1 (PD-1/PD-L1 checkpoint), is one of the major pathways exploited by cancer cells to suppress the immune response. Recent clinical data from monoclonal antibody blockers of the PD-1/PD-L1 pathway indicate that such drugs restore the anti-tumor immunity, with the potential to produce durable clinical responses for patients.^[4–6]^ Herein, we present a case report of a patient with melanoma and a sequence of treatments that lead to disease regression over the past 5 years. We describe tolerance and response to various agents, including nivolumab treatment failure, followed by a surprisingly durable pembrolizumab clinical response.

## 2 Case report

**Figure 1:**
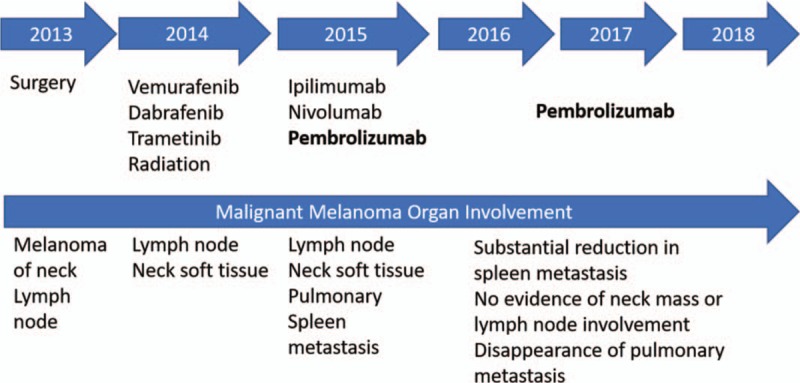
Treatment history. Timeline, therapy, and response to regimen from presentation of metastatic disease to present.

**Figure 2:**
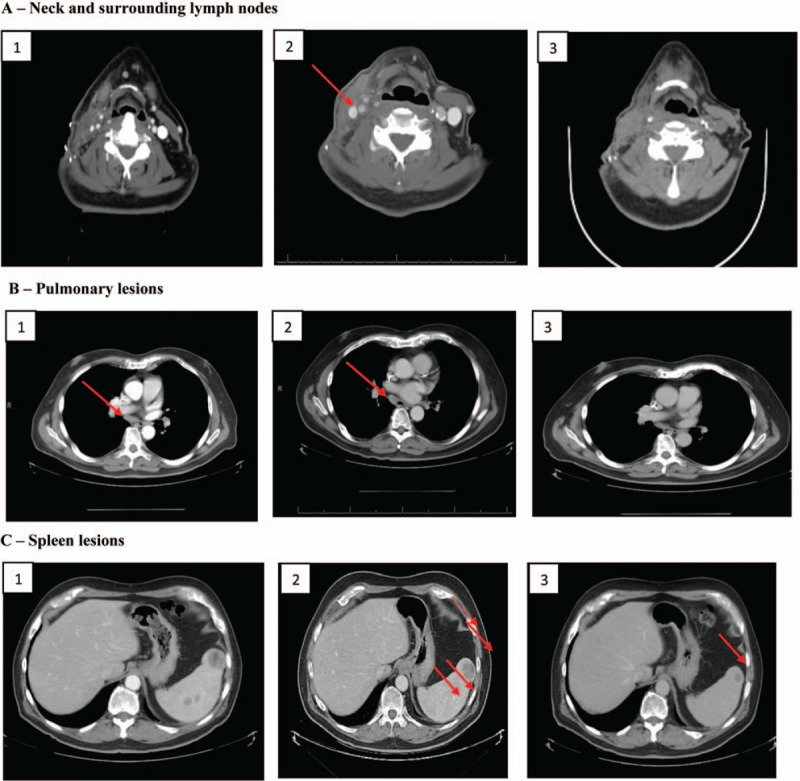
Response to therapy monitored by computed tomography. (A) Neck lesion and surrounding lymph nodes: (1) Before BRAFi treatment (January 2014). (2) Recurrence of melanoma after BRAFi treatment (December 2014). (3) After anti-CTLA-4 treatment (April 2015). (B) Pulmonary lesions: (1) After nivolumab and before pembrolizumab treatment (August 2015). (2) During pembrolizumab treatment (March 2016). (3) During pembrolizumab treatment (April 2018). (C) Spleen lesions: (1) Before pembrolizumab treatment (October 2015, size 3.5 cm). (2) During pembrolizumab treatment. (March 2016, size 3 cm). (3) During pembrolizumab treatment (April 2018, size 1.5 cm).

**Figure 3:**
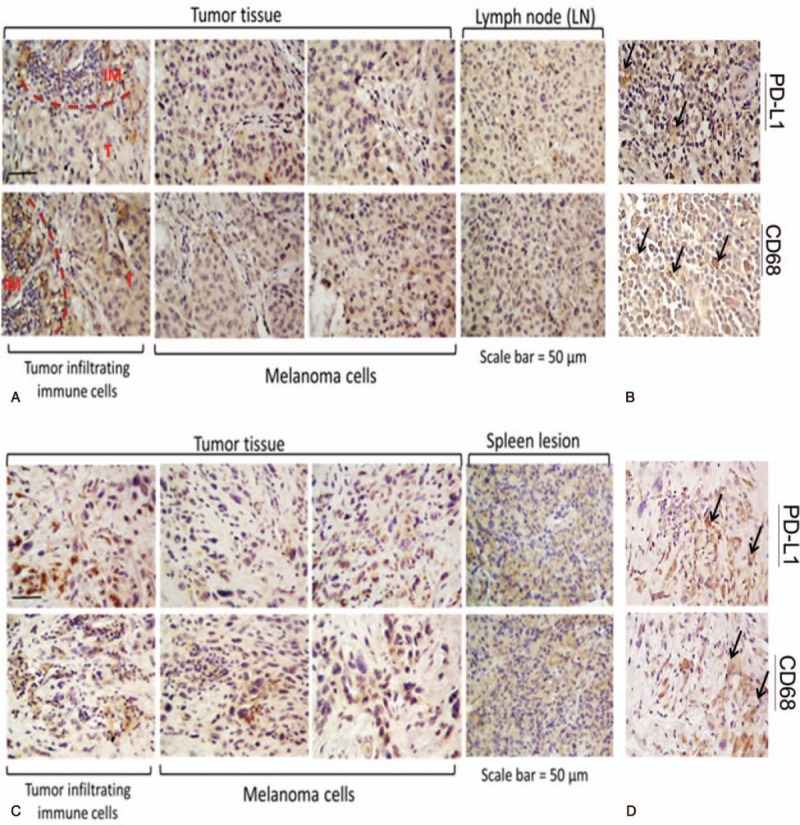
Immunohistochemical staining of the neck, lymph node, and spleen lesions. (A) Visualization of PD-L1 expression in the neck and lymph node lesions *before* combination therapy with *BRAF and MEK inhibitors* (December 3, 2013). The areas circled with red dashed lines indicate the presence of immune cells (IM) adjacent to tumor cells (T). Moderate expression of PD-L1 appears in both immune cells and tumor cells. (B) Colocalization of PD-L1 and macrophage marker CD68 in the neck lesion. Several tumor infiltrating macrophages (black arrows) also express PD-L1. (C) Visualization of PD-L1 expression in the neck and spleen lesions from the same patient *resistant to* a combination therapy with *BRAF and MEK inhibitors* (September 4, 2015). High levels of PD-L1 are present in tumor infiltrating immune cells and tumor cells. (D) Same observation as panel B above, from the 2015 sample of the neck lesion.

The patient written-informed consent was obtained. Patient information was de-identified and the anonymity was maintained. The patient with pathologically confirmed melanoma and BRAF 600E mutation was treated with BRAF and MEK inhibitors, followed by CTLA-4 and PD-1 antibodies. Please refer to Figure 1 for the full treatment history. Computed tomography (CT) and positron emission tomography-computed tomography (PET-CT) scans were utilized to assess response throughout the therapy from 2013 to 2018 (Fig. 2A–C). PD-L1 assays were achieved by immunohistochemistry (Fig. 3A–D). This 76-year-old white male noticed a papular lesion on the right side of his neck and underwent biopsy showing nodular melanoma. Staging CT scans showed no evidence of distant metastatic disease. Therefore, the patient underwent wide local excision and lymph node dissection on December 26, 2013 with the staging as T4bN3M0. The strategy was to provide the patient with adjuvant radiation; however, the patient had a rapidly recurrent disease with dermal infiltration. Repeated CT scan in late January 2014 showed two enlarged lymph nodes in the right side of the neck (Fig. 2A, box 1). Molecular mutation study showed that the tumor harbored BRAF mutation at V600E. IHC staining results also revealed that PD-L1 expression and tumor-infiltrating macrophage marker CD68 appeared in the tumor (Fig. 3A–B) before BRAF and MEK inhibitors treatment in December 2013. PD-L1 levels were moderate in both tumor cells and immune cells (IM, circled with red dash lines) adjacent to tumor cells of a neck skin lesion. Figure 3B displays the colocalization of PD-L1 and CD68 and hence indicates that PD-L1 expression was also expressed on the membrane of tumor-infiltrating macrophages (black arrows).

Due to the rapidity of the disease progression as seen in the CT image from January 2014 (Fig. 2A, box 1), the patient was treated with a BRAF inhibitor, vemurafenib (960 mg twice a day) on February 13, 2014. About 10 days after starting the medication, he was hospitalized for a severe reaction to vemurafenib categorized by fever measuring 102 F, hypertension of 165/79, weakness, maculopapular rash (resembled Steven-Johnson syndrome), tachycardia 110 bpm, pancytopenia, and acute kidney injury with a creatinine of 1.7 with baseline creatinine of 1. He recovered with symptomatic management. Subsequently, treatment was changed to a combination of MEK and BRAF inhibitors, trametinib (2 mg daily) plus dabrafenib (150 mg daily). This combination produced an excellent treatment response. Consolidation with radiation was done, followed by resumption of treatment with dabrafenib and trametinib. However, in December 2014, about 10 months post combination therapy with BRAF and MEK inhibitors, melanoma recurred in the neck (Fig. 2A, box 2). The subsequent biopsy showed malignant epithelioid neoplasm consistent with malignant melanoma, present in the soft tissue of the neck.

Therefore, the treatment was changed to ipilimumab (3 mg/kg every 3 weeks) in February 2015. After the third dose, the patient complained that his neck was stiffer, and he felt that his tumor was growing. After completing four doses of ipilimumab, a CT scan was obtained which showed two new hypermetabolic foci within the right neck (Fig. 2A, box 3) suspicious for neoplasm with interval enlargement of the innumerable pulmonary nodules with associated hypermetabolism, compatible with pulmonary metastases.

Secondary to disease progression, the treatment option was changed to nivolumab **(**3 mg/kg every 2 weeks) in May 2015. After two cycles of nivolumab, the patient complained about lethargy secondary to thyroid dysfunction that improved after treatment with levothyroxine. The patient further expressed the need to chew food very well before he could swallow and therefore felt his tumor mass was increasing in size. Nivolumab was continued on the premise that progression of melanoma may occur before a response would be seen. After nine cycles of nivolumab, CT scans showed a decrease in pulmonary metastasis (Fig. 2B, box 1), but detected the presence of multiple lesions in the spleen (Fig. 2C, box 1) and presence of hilar and mediastinal adenopathy. Subsequently, the splenic mass biopsy was done and immunochemical staining for PD-L1 (clone SP263) was performed by Quest Laboratory (FDA approved). While waiting for the pathology report, the patient completed a total of ten doses of nivolumab.

Immunohistochemical staining of the neck mass and spleen tissue (Fig. 3C–D) showed high levels of PD-L1 present in tumor-infiltrating immune cells and tumor cells (T) that were resistant to BRAF and MEK inhibitor combination treatment (September 2015). Based on the staining for PD-L1 expression of the neck tissue, PD-L1 was more scattered (vs localized) and PD-L1 expression was more intense after resistance to BRAF/MEK inhibitor treatment. As previously mentioned, there was a colocalization of PD-L1 on the membranes of the tumor cells and macrophages in the same area analyzed.

Due to the fact that 40% of metastatic melanoma cells from the splenic lesion expressed PD-L1, we decided to change treatment to pembrolizumab dosed at 2 mg/kg every 3 weeks in October 2015 even though the patient failed nivolumab. Pembrolizumab doses were ultimately changed to a fixed dose of 200 mg every 3 weeks based on prescribing information update. After 8 cycles of pembrolizumab, the patient reported non-specific pain in both legs and a decrease in his ability to walk. The patient is a heavy drinker and therefore it was not clear if his symptoms were associated with drug-induced versus alcoholic neuropathy. The decision was made to continue with therapy with close monitoring of symptoms. The patient further tolerated the treatment well with resolution of hilar and mediastinal adenopathy and slow resolution of spleen metastasis (tumor size regression from 3.5 to 2.7 and 1.5 cm). Please refer to Figure 2B (boxes 2–3) for CT imaging showing complete pulmonary lesions disappearance after pembrolizumab initiation. Figure 2C (box 2) also shows the disappearance of most spleen lesions after about 5 months of pembrolizumab treatment. Currently, the patient has only one solitary splenic lesion remaining (Fig. 2C, box 3). As of July 2018, the patient has received and tolerated 33 months of treatment with pembrolizumab and a final radiofrequency ablation (RFA) procedure is planned.

## 3 Discussion

### 3.1 BRAF and MEK inhibitors

BRAF mutations are seen in around 50% of patients with melanoma.^[1,7]^ Response rates to BRAF/MEK inhibitor combination therapy approaches 70% in patients with metastatic melanoma.^[8–10]^ When compared with either single-agent dabrafenib or vemurafenib, the combination of dabrafenib plus trametinib improves response rate, the duration of response, a progression-free survival, and the overall survival.^[8,9]^ In our case, the patient did not tolerate vemurafenib monotherapy. Vemurafenib adverse effects include: skin rash (grade 3: 7–8%), skin photosensitivity (grade 3: 3%), pruritus (grade 3: 2%), maculopapular rash (grade 3: 2–6%), xeroderma, erythema, papular rash, increased serum creatinine (up to 3 times upper limit of normal (ULN): 26%; greater than 3× ULN: 1%).^[11,12]^ During the treatment combination with trametinib and dabrafenib, the patient was monitored closely for tolerance and potential drug–drug interactions secondary to previous intolerance to BRAFi. In addition, both vemurafenib and dabrafenib contain sulfonamide groups and there was a concern for cross-reactivity. It is known that cross-reactivity between BRAFi and sulfonamide compounds have been reported in allergic patients.^[13]^ Half of the patients treated with BRAF-targeted monotherapy usually relapse within 6 months of treatment initiation, due to development of drug resistance.^[14–16]^ Our patient progressed after an anticipated duration of response of less than 1 year.^[8,9]^

### 3.2 Immune checkpoint inhibitors

Since the response to ipilimumab could be significantly delayed and melanoma could progress before any improvement could be observed,^[17,18]^ the patient completed the fourth dose of ipilimumab as scheduled. While the patient had no significant adverse effect from ipilimumab, the treatment was changed to anti-PD1 antibody, nivolumab, secondary to disease progression as evidenced by patient's symptomology and radiographic imaging (Fig. 2A, box 3).

Between 25% and 40% of patients diagnosed with melanoma have responded to pembrolizumab or nivolumab.^[19,20]^ Importantly, a recent report of a large cohort of patients with metastatic melanoma who achieved complete response when treated with pembrolizumab showed that a continued remission may persist even after discontinuing pembrolizumab.^[6]^ Presumably, patients may be cured of the disease. Nivolumab and pembrolizumab possess a similar mechanism of action. However, no head-to-head comparison of these drugs’ efficacy has been reported to date. Importantly, there is also no comprehensive study that has evaluated the likelihood for patients to respond to secondary checkpoint inhibitors when an initial treatment with either nivolumab or pembrolizumab failed, and whether this therapeutic strategy might be worth testing in a trial. Herein, we reported a case which clearly demonstrated that responding to pembrolizumab is still possible even after the patient failed both ipilimumab and nivolumab.

Today, both PD-1 inhibitors are approved by the FDA for the treatment of melanoma; However, in 2015, the only PD-1 blocker available was nivolumab. Treatment with nivolumab was relatively well-tolerated by our patient, apart from hypothyroidism requiring an additional treatment with levothyroxine. It is well-known that an increase of the tumor size could be observed during the course of an immunotherapy treatment due to a delay in response (around 3 months).^[18]^ Therefore, we continued to treat our patient with nivolumab, despite disease progression, for a total of ten cycles at which point, a mixed response was observed. Indeed, pulmonary nodules have disappeared, but the patient developed multiple metastatic lesions in the spleen (Fig. 2C, box 1). The subsequent biopsy showed that 40% of the spleen tumor cells were positive for PD-L1 (Fig. 3C–D). Furthermore, we also documented high PD-L1 in tumor-infiltrating macrophages. Based on these results, we decided to treat the patient with pembrolizumab despite nivolumab treatment failure. To our delight, all sites of metastasis have responded to the pembrolizumab treatment with no lesions seen in the neck, mediastinum, and only one lesion remaining in the spleen. Moreover, the patient has not relapsed or developed any new lesions for the past 2 years.

Currently, there are no established biomarkers to predict response to checkpoint inhibitors besides PD-L1 positivity and high mutation load. PD-L1 is an inducible molecule and tumors are frequently heterogeneous. Therefore, with as little as 1% of cell expression being considered positive, it is uncertain how reproducible a particular assay would be even in an individual patient's tumor. In addition, discordance between primary tumor and metastases for PD-L1 positivity has been observed. Nonetheless, our patient is experiencing excellent and durable melanoma treatment response since the treatment initiation with pembrolizumab. The rationale behind such a response remains to be answered. To this aim, we searched for the possible reasons and propose some potential explanations for the pharmacodynamic differences between these two monoclonal antibodies.

### 3.3 Structural biology of PD-1 blockers: a comparative analysis

It is commonly accepted that PD-L1 (*i.e.* CD274, B7-H1) precludes autoimmunity by engaging to the PD-1 receptor (*i.e.* CD279) on activated T cells. PD-1 is a monomeric type I surface glycoprotein with an immunoglobulin (Ig) superfamily topology (CD28 receptor family) consisting of a single V-set domain (IgSF) attached to a transmembrane and an intracellular domain. Upon binding to PD-L1, PD-1 initiates an inhibitory signaling cascade triggered by an immunoreceptor tyrosine-based inhibitory motif (ITIM) and immunoreceptor tyrosine-based switch motif (ITSM) inside the intracellular signaling domain. Given that the PD-1/PD-L1 immune checkpoint is initiated by ligand–receptor interactions, this recognition can be readily inhibited by mAbs to restore the anti-tumor immunity. PD-1 antibodies (or blockers) such as pembrolizumab and nivolumab are the current flagships of immunotherapeutic therapies, which have demonstrated an important efficacy in melanoma.^[21]^

Despite the recent impressive clinical results of PD-1 blockers, we know relatively little about the structure and interactions of human PD-1 with its endogenous ligand PD-L1 or with PD-1 blockers (pembrolizumab and nivolumab).^[22]^ Given the significant differences in response induced by nivolumab and pembrolizumab reported in the patient case herein, we decided to assess the ligand–receptor interactions that are likely responsible for the pharmacodynamic differences between these medications.^[24]^ Even though recent studies have reported important steps in understanding PD-1–PD-L1 interactions,^[23–25]^ it is only recently that several crystal structures of the extracellular V-domain of human PD-1 either in complex with PD-L1 (protein data bank or PDB code: 4ZQK) or with the antigen-binding fragments (Fabs) of pembrolizumab (PDB code: 5GGS) and nivolumab (PDB code: 5GGR) have been reported.^[26–28]^ Therefore, a side by side comparison of the blocker binding models and of PD-L1 complexed with PD-1 is proposed to evaluate the main structural “hot” spots and epitopes of PD-1, as well as the key conformational changes imparted to PD-1 by the blockers upon binding events.

**Figure 4:**
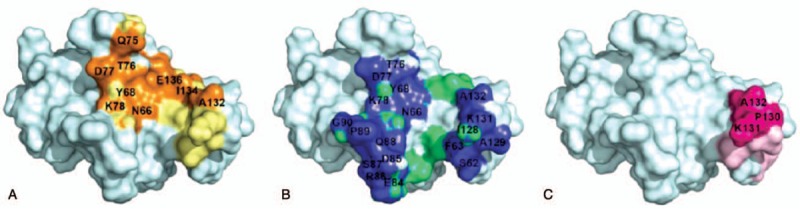
Surface representation of PD-1 binding interfaces: (A) with PD-L1: orange and yellow represent key hydrophilic and hydrophobic residues, respectively. (B) with pembrolizumab: blue and light green represent key hydrophilic and hydrophobic residues respectively. (C) with nivolumab: magenta and light pink represent key hydrophilic and hydrophobic residues respectively.

A detailed mapping of PD-1 molecular interactions with ligands: PD-L1 as well as with pembrolizumab and nivolumab Fabs is reported in Tables S1 and S2 (see Supplemental Content) and graphically represented in Figure 4. Overall, PD-1 shares similar sites of interactions (epitopes) in the complex with PD-L1 or pembrolizumab (see Tables S1 and S2, Supplemental Content). Indeed, as shown in the PD-1–pembrolizumab interface (Fig. 4B), the patches of residues from the PD-1 *β*-strand C, the neighboring CC’ loop and the FG loop promote many hot spots of interactions to the pembrolizumab heavy chain. In addition, an important binding domain (not present in the PD-1–PD-L1 complex) is involved in the interaction of pembrolizumab heavy chain complementary determining region (HCDR3) with the C’D loop [P83-R94] of PD-1. This patch of seven “hot" residues from the C’D loop is also extremely important to ensure a maximum of hydrophobic interactions between pembrolizumab and PD-1 (see Table S2, Supplemental Content). In contrast, while some binding interactions between the FG loop of PD-L1 and nivolumab also exist at the PD-1–nivolumab interface (Fig. 4C), the main hydrophobic interactions (and two H-bonds) are localized from the heavy chain of nivolumab to the tip of the floppy and relatively unstructured *N*-terminal region of PD-1: the N loop residues [S27-R30]. Taken together, this comparative binding analysis suggests that PD-L1 and pembrolizumab bind similarly to the CC’ loop, flanked by the neighboring *β*-strand C, and the distal FG loop of PD-1. The higher affinity of pembrolizumab might likely arise from a unique and large patch of interactions engaging the C’D loop of PD-1 thus forcing an important motion across the receptor.

### 3.4 Dynamics of binding to PD-1

Furthermore, morphing calculations were achieved using PyMol^[29]^ to assess the level of conformational changes induced upon ligand or blockers approach and binding to PD-1. The morphing movies display an extrapolation of the conformational motion between the crystal structure apo-PD-1 in a free form (PDB code: 3RRQ) and its bounded counterpart to the corresponding ligands (PD-L1: link, pembrolizumab: link, nivolumab: link, see videos from Supplemental Videos 1–3 respectively). In these morphing animations, the PD-1 backbones are represented in a ribbon form to examine the level of superimposition throughout the motion of a putative binding approach. The color-coded level of superimposition is presented in a gradient scale as follows: blue for the minimum pairwise root-mean-square deviation (RMSD) or best fit, red indicating a higher RMSD, and gray indicating unaligned residues. During the PD-L1–PD-1 complex formation (see video, Supplemental Video 1: link), both CC’ loop, flanked by the neighboring *β*-strand C, and the distal FG loop are shown to close down onto the ligand, supporting that these domains are important epitopes of PD-1. Interestingly, the *β*-strand C also twists upon binding promoted by a conformational flip of the three final residues _PD1_R69-_PD1_M70-_PD1_S71 leading to an important restructuration of the CC’ loop [_PD1_P72-_PD1_T76] into a 3_10_-helix when bounded. A similar motion of PD-1 is also observed during the pembrolizumab–PD-1 complex formation (see video, Supplemental Video 2: link). Additionally, upon binding to pembrolizumab, the PD-1 receptor undergoes an important conformational change in the second epitope: the FG loop domain, which ultimately enables the _PD1_K131–_LC_E59 salt bridge to take place. Finally, the third animation clearly demonstrates that nivolumab–PD-1 binding operates with a drastically different mode through the saggy *N*-terminal N loop and primarily the FG loop region. In this case, to the FG loop region of PD-1 (_PD1_P130, _PD1_K131 and _PD1_A132) inserts into the antigen-binding cleft of nivolumab without any important conformational change via a characteristic lock-and-key mechanism (see video, Supplemental Video 3: link).

## 4 Conclusion

To the best of our knowledge, there has been no report of a clinical comparison between pembrolizumab and nivolumab efficacy. The present case of a patient with malignant melanoma provides evidence of an unexpected and durable treatment response to pembrolizumab after a nivolumab treatment failure. Pharmacologic differences between these inhibitors as well as variations of immune cells in the tumor microenvironment could potentially offer an explanation for the response to pembrolizumab therapy.^[21]^ The side-by-side binding mode comparison of PD-1 with PD-L1 and the two clinically relevant blockers pembrolizumab and nivolumab shed light into some important differences of protein–protein interaction at the molecular level. This study supports that pembrolizumab binds to the PD-1 immunoreceptor in a competitive fashion to PD-L1 and its high affinity is mainly attributed to the HCDR3 loop which binds to both C’D and FG loop epitopes of PD-1. More importantly, the evaluation of dynamic binding by conformational morphing suggests for the first time that pembrolizumab induces a pincer effect on PD-1 accompanied by a significant change in conformation of the *β*-strand C. Further studies will be needed to understand the full outcome of conformational changes of the *β*-strand C imparted to the PD-1 protein upon binding and to understand how this response might alter the phosphorylation cascades in the cytoplasmic ITIM and ITSM domains of PD-1 which are responsible to recruiting SHs2 domain-containing phosphatases to induce immunosuppression activity. The present study does not exclude that PD-1 blockers (*e.g.* nivolumab) might also interact with other immunoreceptors of the CD28/CTLA-4 family to achieve their clinical efficacy.

## Acknowledgments

We thank Joy Garcia, charge-nurse from the oncology and hematology department at the Bruce W. Carter VA Medical Center for her care of the patient throughout the treatment course.

## Author contributions

**Conceptualization:** Tanja Lepir, Niramol Savaraj.

**Data curation:** Tanja Lepir, Niramol Savaraj.

**Formal analysis:** Stéphane P. Roche, Niramol Savaraj.

**Investigation:** Stéphane P. Roche, Mehdi Zaghouani, Ying-Ying Li.

**Methodology:** Stéphane P. Roche, Niramol Savaraj.

**Resources:** Tanja Lepir, Ying-Ying Li, Miguel Suarez.

**Software:** Mehdi Zaghouani.

**Supervision:** Stéphane P. Roche, Niramol Savaraj.

**Validation:** Niramol Savaraj.

**Visualization:** Tanja Lepir, Miguel Suarez, Maria Jose Irias, Niramol Savaraj.

**Writing – original draft:** Tanja Lepir.

**Writing – review & editing:** Tanja Lepir, Stéphane P. Roche, Maria Jose Irias, Niramol Savaraj.

## Supplementary Material

Supplemental Digital Content

## Supplementary Material

Supplemental Digital Content

## Supplementary Material

Supplemental Digital Content

## Supplementary Material

Supplemental Digital Content
