# Supplementary material for: Nivolumab to pembrolizumab switch induced a durable melanoma response: A case report
Source: Medicine (Baltimore). 2019 Jan 11;98(2):e13804. doi: 10.1097/MD.0000000000013804 (PMC6336603; doi:10.1097/MD.0000000000013804)
Supplement: Supplemental Digital Content [file medi-98-e13804-s001.docx]

Supplemental Content

Nivolumab―Pembrolizumab Switch

Induced a Durable Melanoma Response: A Case Report

Tanja Lepir,* Mehdi Zaghouani, Stéphane P. Roche, Ying-Ying Li, Miguel Suarez, Maria-Jose Irias and Niramol Savaraj

**Comparative Analysis of PD-1 Interfaces with PD-L1, Nivolumab and Pembrolizumab**

A side by side comparison of the blocker binding models and of PD-L1 complexed with PD-1 is proposed to evaluate the main structural “hot” spots and epitopes of PD-1, as well as the key conformational changes imparted to PD-1 by the blockers upon binding events (Tables S1 and S2).

Ligand–receptor interactions have been cross-analyzed using PyMol and LigPlot+ software with thresholds of 2.7 Å for hydrogen bonds (H-bonds) and bridged H-bond relayed by water molecules, 3.35 Å for salt bridges and 2.9-3.9 Å for hydrophobic van der Waals interactions. ^[SI^^[[1]](#endnote-1)^^, SI^ ^[[2]](#endnote-2)]^ A detailed mapping of PD-1 molecular interactions with ligands: PD-L1 as well as with pembrolizumab and nivolumab Fabs is reported in Tables S1-S2 and graphically represented in Figure 4. In the Tables below, PD-1 residues reported in green have been found to bind to a least two ligands and residues in blue to all three ligands. Given the significant strength of salt bridges in protein–protein interactions (PPI),^[SI^^[[3]](#endnote-3), SI^^[[4]](#endnote-4)]^ it is relevant to notice that PD-1 interacts with PD-L1 through two ionic bridges _PD1_D77 with _PDL1_K124 and _PD1_E136 with _PDL1_R113 (Table S1). Similarly, ionic bridges between PD-1 and pembrolizumab are formed by _PD1_D85 with _HC_R99 and _PD1_K131 with _LC_E59/_HC_D108 respectively, while nivolumab does not create such interaction. In regards to H-bond interactions in the PD-1–PD-L1 complex, large patches of residues are localized on the *β*-strand C [F63-M70], the neighboring CC’ loop [S71-T76] and a few more residues towards the end of the FG loop [L128-I134] of PD-1. Interestingly, very similar regions of PD-1 are shown to be engaged in hydrophobic interactions with PD-L1 through two large patches of residues from the *β*-strand C, the CC’ loop and the FG loop (Table S2). Overall, PD-1 shares similar sites of interactions (epitopes) in complex with PD-L1 and pembrolizumab (Tables S1-S2). Our analysis supports that in the PD-1–pembrolizumab interface, the patches of residues from the PD-1 *β*-strand C, the neighboring CC’ loop and the FG loop promote many hot spots of interactions to the pembrolizumab heavy chain. In addition, an important binding domain (not present in the PD-1–PD-L1 complex) is involved in the interaction of pembrolizumab complementary determining region (HCDR3) with the C’D loop [P83-R94] of PD-1. This patch of seven “hot” residues from the C’D loop is also extremely important to ensure a maximum of hydrophobic interactions between pembrolizumab and PD-1 (Table S2). In contrast, while some binding interactions between the FG loop of PD-L1 and nivolumab also exist at the PD-1–nivolumab interface, the main hydrophobic interactions (and two H-bonds) are localized from the heavy chain of nivolumab to the tip of the floppy and relatively unstructured *N*-terminal region of PD-1: the N loop residues [S27-R30]. Accordingly to this comparative analysis, _PD1_L128, _PD1_K131 and _PD1_A132 are important residues that interact with each ligand studied. Other key residues of PD-1 involved in hydrogen binding are: _PD1_N66, _PD1_T76, _PD1_D77, _PD1_K78, _PD1_D85 and _PD1_E136.

**Table S1.** Summary of key polar interactions from residues at the surface of contact between PD-1 with the natural ligand PD-L1 or with pembrolizumab and nivolumab. *^a,b^*

| **PD-1** | **PD-L1**  **[PDB: 4zqk]** | **Pembrolizumab [PDB: 5ggs]** | | **Nivolumab [PDB: 5ggr]** | |
| --- | --- | --- | --- | --- | --- |
|  |  | C (HC) | D (LC) | A (HC) | B (LC) |
| **P28^†^** | - | - | - | Y53^†^ | - |
| **R30^‡^** | - | - | - | N31^†^ | - |
| **S62^‡^** | - | - | Y57^‡^ | - | - |
| **F63^†^** | - | - | Y34^‡^ | - | - |
| **N66^‡^** | A121^†^ | R102^†^ | - | - | - |
| **Y68^‡^** | D122^‡^, Y123^†^ | - | - | - | - |
| **Q75^‡^** | D26^‡^, R125^†^ | - | - | - | - |
| **T76^‡†^** | Y123^†^, K124^‡^ | Y101^‡^ | - | - | - |
| **D77^‡^** | K124^‡^ | - | - | - | - |
| **K78^‡†^** | F19^†^, A121^†^ | Y33^‡^, N52^‡^*, Y101^†^ | - | - | - |
| **E84^†^** | - | - | S95^†^* | - | - |
| **D85^‡^** | - | R99^‡^*, D104^†^* | - | - | - |
| **R86^†^** | - | N59^‡^* | S95^†^* | - | - |
| **S87^†‡^** | - | Y35^‡^, N59^‡^, R99^‡^ | S95^†‡^* | - | - |
| **Q88^†‡^** | - | N52^‡^*, N59^‡^* | - | - | - |
| **P89^†^** | - | N52^‡^* | - | - | - |
| **G90^†^** | - | T58^†^ | - | - | - |
| **L128^†^** | - | - | Y53^‡^ | - | - |
| **A129^†^** | - | - | Y53^‡^*, L58^†^* | - | - |
| **P130^†^** |  | - | - | - | T56^†^ |
| **K131^‡†^** | - | M105^†^,D108^‡^ | E59^‡^ | N99^‡†^, D100^†^ | - |
| **A132^†^** | Q66^‡^ | R102^‡^ | - | - | Y49^‡^ |
| **I134^†^** | Y56^‡^* | - | - | - | - |
| **E136^‡^** | R113^‡^, Y123^‡^ | - | - | - | - |

Residues interactions are marked with ^†^ for backbone, ^‡^ for side chain, and * through a water molecule (HC: heavy chain, LC: light chain). *^b^* PD-1 residues highlighted in blue engaged in interactions with the three ligands (PD-L1 and the two blockers), and PD-1 residues highlighted in green engaged with at least two ligands (A = Alanine; D = Aspartic acid; E = Glutamic acid; F = Phenylalanine; G = Glycine; I = Isoleucine; K = Lysine; L = Leucine; M = Methionine; N = Asparagine; P = Proline; Q = Glutamine; R = Arginine; S = Serine; T = Threonine; Y = Tyrosine).

PDB = protein data bank; PD-1 = programmed cell death receptor-1; PD-L1 = programmed death ligand 1

**Table S2.** Summary of key hydrophobic van der Waals interactions from the surface of contact of PD-1 with the natural ligand PD-L1 and the mAb blockers pembrolizumab and nivolumab. *^a,b^* HC: heavy chain; LC: light chain

| **PD1** | **PD-L1**  **[PDB: 4zqk]** | **Pembrolizumab [PDB: 5ggs]** | | **Nivolumab [PDB: 5ggr]** | |
| --- | --- | --- | --- | --- | --- |
|  |  | C (HC) | D (LC) | A (HC) | B (LC) |
| **S27** | - | - | - | W52 | - |
| **P28** | - | - | - | W52, Y53, N99 | - |
| **D29** | - | - | - | N31, G33, Y53, N99, D100 | - |
| **R30** | - | - | - | N31, Y53 | - |
| **E61** | - | - | - | T28 | - |
| **S62** |  | - | Y57 | - | - |
| **F63** | - | - | Y34 | - | - |
| **V64** | - | F103 | Y34 | - | - |
| **N66** | A121 | R102 | - | - | - |
| **Y68** | D122, Y123 | R102, Y101 | - | - | - |
| **N74** | R125 | - | - | - | - |
| **Q75** | V23, D26, K124, R125 | - | - | - | - |
| **T76** | Y123, K124 | Y101 | - | - | - |
| **K78** | F19, A121, D122 | Y33, Y101, R102 | - | - | - |
| **P83** | - | F103 | Y34, Y36 | - | - |
| **D85** | - | R99 | - | - | - |
| **R86** | - | - | R96 | - | - |
| **S87** | - | Y35, N59 | - | - | - |
| **Q88** | - | Y33, N59 | - | - | - |
| **P89** | - | Y33, G50, I51, N52, G57, T58, N59 | - | - | - |
| **G90** | - | G57, T58 | - | - | - |
| **G124** | Y123 | - | - | - | - |
| **I126** | M115, Y123 | - | - | - | - |
| **L128** | I54, M115, S117 | - | Y34, Y53, L54 | Y102 | - |
| **A129** | - | - | - | D100, Y102 | T56 |
| **P130** | - | - | - | D100, D101, Y102 | Y49, A55, T56 |
| **K131** | Q66 | M105 | Y53, E59 | N99, D100 | L46, Y49 |
| **A132** | Y56, Q66 | R102 | - | - | Y49, T56 |
| **I134** | E58, R113, Y123 | R102 | - | - | - |
| **E136** | R113, Y123 | - | - | - | - |

*^a^* Residues interactions are marked with ^†^ for backbone, ^‡^ for side chain, and * through a water molecule. *^b^* Residues highlighted in blue are characterized by making interactions between PD-1 with PD-L1 and the two blockers; residues highlighted in green with at least two ligands (A = Alanine; D = Aspartic acid; E = Glutamic acid; F = Phenylalanine; G = Glycine; I = Isoleucine; K = Lysine; L = Leucine; M = Methionine; N = Asparagine; P = Proline ; Q = Glutamine; R = Arginine; S = Serine; T = Threonine; V = Valine; W = Tryptophan; Y = Tyrosine). PDB= protein data bank; PD1 = programmed cell death receptor-1; PD-L1 = programmed death ligand 1;

1. [SI-] Wallace AC, Laskowski RA, Thornton JM. LIGPLOT: A program to generate schematic diagrams of protein-ligand interactions. *Protein Eng* 1995; 8: 127-34. [↑](#endnote-ref-1)
2. [SI-] The PyMOL Molecular Graphics System, Version 2.0 Schrödinger, LLC. [↑](#endnote-ref-2)
3. [SI-] Horovitz A, Serrano L, Avron B, et al. Strength and cooperativity of contributions of surface salt bridges to protein stability.  *J Mol Biol* 1990; *216*: 1031-44. [↑](#endnote-ref-3)
4. [SI-] Xu D, Tsai CJ, Nossinov R. Hydrogen bonds and salt bridges across protein-protein interfaces. *Protein Eng.* 1997; 10: 999-1012. [↑](#endnote-ref-4)
